# What can the Radiologist do to Help the Surgeon Manage Shoulder Instability?

**DOI:** 10.5334/jbr-btr.1227

**Published:** 2016-11-19

**Authors:** Nicole Pouliart, Seema Doering, Maryam Shahabpour

**Affiliations:** 1 Dept of Orthopaedics and Traumatology, VUB (Vrije Universiteit Brussel) – Universitair Ziekenhuis Brussel (UZ Brussel) Laarbeeklaan 101, B-1090 Brussel, Belgium; 2 Dept of Radiology, VUB (Vrije Universiteit Brussel) – Universitair Ziekenhuis Brussel (UZ Brussel) Laarbeeklaan 101, B-1090 Brussel, Belgium

**Keywords:** Shoulder, Glenohumeral, Dislocation, Instability, Imaging

## Abstract

Imaging of the shoulder forms an important adjunct in clinical decision making in patients with shoulder instability. The typical lesions related with classic anterior and anteroinferior shoulder dislocation are an anteroinferior labral avulsion with or without bony fragment of bone loss – a (bony) Bankart lesion – and a posterolateral humeral head impaction fracture – the Hill-Sachs lesions. These are relatively straightforward to identify on imaging, although normal variants of the inferior labrum and variants of labral damage may cause confusion. Other capsuloligamentous lesions, often associated with less typical types of instability, are much more difficult to identify correctly on imaging, as they occur in the anterosuperior part of the glenohumeral joint with its many normal variants or because they result in more subtle, and therefore easily overlooked, changes in morphology or signal intensity. This paper aims at describing the appearance of the normal and pathologic glenohumeral joint related to shoulder instability. Ample reference will be given as to why identification of abnormalities, whether normal or pathologic, is important to the surgeon facing a treatment decision.

## Introduction

History and physical examination of a patient should identify the type of shoulder instability and the anatomical area likely to be responsible. Traumatic anterior shoulder instability is relatively straightforward to identify. It is usually associated with anteroinferior lesions of the labrum and/or glenoid rim as well as a Hill-Sachs lesion. However, in more severe cases or after numerous dislocations extension to the superior and/or posterior labrum may occur. Humeral avulsions of the glenohumeral ligaments, although rare, need to be excluded. Posterior and anterosuperior instability are distinctly different entities that are more difficult to detect clinically as well as radiologically. Additionally, associated damage of the rotator cuff, the biceps pulley or the cartilage can occur.

Imaging is used to determine whether the surgeon will be faced with a simple Bankart lesion or with lesions that demand a different surgical approach or have an influence on the prognosis. The shoulder surgeon therefore requires precise qualitative and quantitative information on individual components of the glenohumeral joint.

## The Labrum

### What does a normal labrum look like?

The labrum is a fibrous ring that surrounds the glenoid rim, joined with the origin of the long tendon of the biceps superiorly and that of the triceps inferiorly. Usually, the labrum is in continuity with the articular cartilage as well as the glenohumeral ligaments (GHL).

For most of its circumference, it commonly is triangular (anterior 45%, posterior 73%) or round (anterior 19%, posterior 12%) in shape. However, infrequent variations of the anterior labrum include a cleaved or notched appearance, flattening or even absence. When seen posteriorly, this is suggestive of a tear. A notched labrum is often associated with redundant anterior or posterior capsule or may be due to folding of the middle and inferior GHL due to internal rotation of the humerus. In contrast, external rotation may result in a more flat labrum. A thinner or even absent labrum may be an age-related degenerative change, but can also be associated with instability [1,2,3,4,5,6,7,8,9,10,11,12,13].

### Can we differentiate normal wear from pathology?

According to several studies, Magnetic Resonance (MR) abnormalities (79%) and even labral tears (36 to 62%) can be found in both the throwing and non-throwing shoulders of asymptomatic high-level athletes.

**Figure 1:**
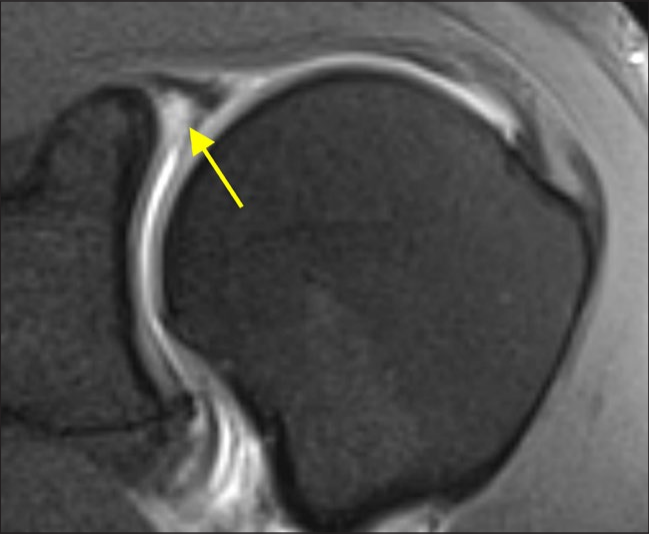
**Degenerative labrum.** On coronal fat-saturated T1 weighted MR arthrographic image, degenerative changes in the labrum may appear as increased signal intensity. A hyperintense signal in the labrum superiorly (arrow) at the labrobicipital complex can also be considered as a type I SLAP lesion.

Acute as well as repetitive trauma may damage the labrum. The labrum may, however, display degenerative changes from as early as the third decade. This will be visible as changes in signal intensity on MR images (Figure 1) and may make interpretation more difficult. Although the labrum usually has low signal intensity on MRI, a normal labrum can display areas of increased signal intensity, especially on intermediate-weighted sequences that can be linear as well as rounded in shape. In the posterosuperior labrum a higher signal intensity may be due to the magic-angle effect, shoulder positioning or specific Echo Time (TE) parameters [3,5,7,8,11,14,15,16,17,18,19,20,21,22,23,24,25].

### The labrum is firmly attached to, and flush with the glenoid rim, isn’t it?

#### The labrum can overlap with the cartilage

Inferiorly, the labrum partially extends up to 5 mm over the glenoid rim and, therefore, overlaps the articular cartilage. This undercutting of the cartilage with its linear intermediate signal intensity should not be mistaken for a labral tear. Anterosuperiorly, there usually is little or no overlap [3,26,27].

#### What are the signs of normal superior variants (11 to 1 o’clock)? How can we differentiate them from pathology?

**Figure 2:**
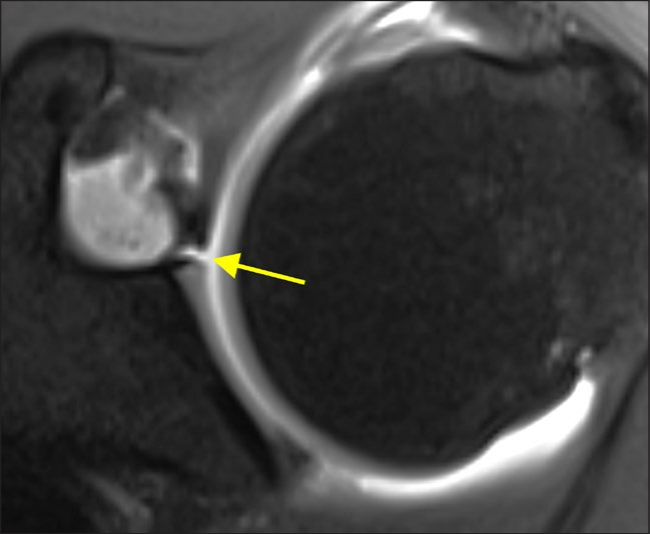
**Anterosuperior labral recess.** On axial fat-saturated T1 weighted MR arthrographic image, an anterosuperior labral recess (arrow) is seen. It is a normal anatomic variant of glenoid labrum. It can be differentiated from a pathologic labrum by its medially oriented signal, smooth edges, a width of less than 2 mm and normal adjacent labral signal.

The superior labrum, between 11 and 1 o’clock, blends with a large portion of fibers from the tendon of the long head of the biceps and is prone to variation in its attachment to the glenoid rim. The labrum can be firmly attached and flush with the cartilage; it can be attached more medially with undercutting of hyaline glenoid articular cartilage and a relatively shallow superior recess (sulcus), or it can be overhanging the glenoid rim with a projecting meniscoid appearance and a deep undercutting recess. The superior recess is quite common, appearing in up to 75% of patients, increasing in frequency with age, and has an average length of cartilage of 3 to 4 mm. On coronal oblique fat-saturated T1-weighted MR or CT arthrographic images, the sublabral recess can be differentiated from a type II superior labral anteroposterior (SLAP) lesion on the basis of orientation and extension of the contrast-outlined linear structure. Normal articular cartilage undercutting and a superior recess are characterized by a medially oriented hyperintensity that follows the bone (linear inferiorly, curved superioly) with smooth edges, a width < 2 mm, and normal adjacent labral signal (Figure 2). Anterior and inferior, this signal usually does not extend throughout the labrum, although full extension has also been described. A normal superior recess is confined to the 11 and 1 o’clock position. Although articular hyaline cartilage has a high signal intensity on axial gradient echo and fat-saturated MR images, its signal intensity is less than that of fluid as well as that of intraarticular contrast material. Findings suggestive of a SLAP tear include a high signal intensity that is globular, irregular or laterally curved into the substance of the labrum on coronal oblique images, an anterior to posterior linear hyperintense signal on axial T1-weighted MR arthrography (MRA), a signal of irregular width measuring > 2 mm. Because contrast material or joint fluid will extend under the free edge of a meniscoid-type superior labrum, it may be mistaken for a labral tear [1,3,4,11,14,21,25,26,28,29,30,31,32,33,34,35,36,37,38,39,40,41,42,43,44,45,46,47,48,49].

#### What are the signs of normal anterosuperior variants (1 to 3 o’clock)? How can we differentiate them from pathology?

**Figure 3:**
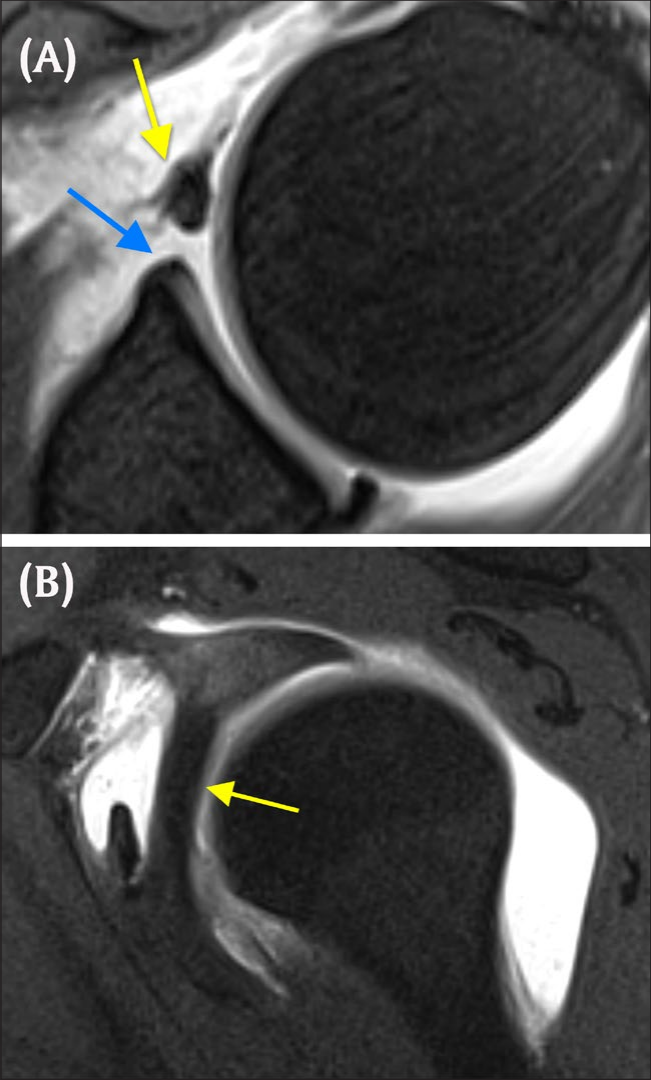
**Buford complex. A.** Axial fat-saturated T1 weighted MR arthrographic image shows the absence of the anterosuperior labrum (blue arrow). A globular hypointense oval mass is seen anterior to the defect (yellow arrow). This is a thickened middle glenohumeral ligament, part of Buford complex and an example of ‘GLOM’ – glenoid labrum ovoid mass. **B.** The thickened middle glenohumeral ligament is displayed on sagittal fat saturated T1 weighted MR arthrographic image as a thick band (yellow arrow) running behind the subscapularis tendon.

For the anterosuperior segment of the labrum between 1 and 3 o’clock, three common variants have been described. A small anterosuperior recess can occur anterosuperiorly, usually as an extension of the superior sulcus to 2 o’clock. The sublabral foramen (or hole) occurs when an intact anterosuperior labrum is not attached to the glenoid rim, typically between 1 and 3 o’clock, and has a prevalence of 11% to 21%. The middle GHL usually remains attached to this loose labrum and may appear either flat or sheet-like or thickened and cord-like. In a Buford complex (Figure 3), a cordlike MGHL is associated with absence of the corresponding anterosuperior labrum and has an incidence of 7.5 to 15%. The sublabral foramen and the Buford complex are best seen on axial fat saturated T1-weighted MRA. The thickened middle glenohumeral ligament can also be visualised on sagittal oblique T1-weighted MRA. Although these variants are usually restricted to the anterosuperior area, extension into the anteroinferior labrum has been noted making it difficult to differentiate from a Bankart lesion. These anterosuperior variants may also predispose to superior labral lesions. Differentiating these normal variants from pathology is crucial because fixing the normal labrum and/or the middle glenohumeral ligament will result in loss of external rotation and surgical failure [1,3,9,14,25,30,32,33,34,35,36,37,38,39,40,44,46,50,51,52,53,54,55,56,57,58,59,60,61,62,63,64].

#### Are there variants in the other areas?

**Figure 4:**
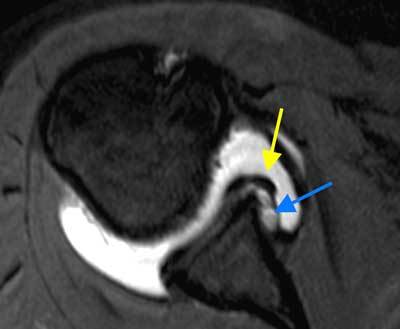
**Paralabral cyst.** On axial fat-saturated T1 weighted MR arthrographic image, the identification of a paralabral cyst (blue arrow) can help differentiating a pathologically torn labrum (yellow arrow) from normal anatomic variants of labrum.

In the anterior, anteroinferior, and posterosuperior labrum, shallow clefts or grooves of <1 mm can be identified in up to 40% of normal labra. Recesses that are slightly deeper (2–3 mm) are much less common and secondary signs of instability, such as a paralabral cyst (Figure 4), or an associated Hill-Sachs lesion may help in differentiating pathology from a normal variant [38,58,65,66].

## What shouldn’t we miss in patients with recurrent anterior dislocations?

### Capsulolabral lesions: not only the typical Bankart lesion

**Figure 5:**
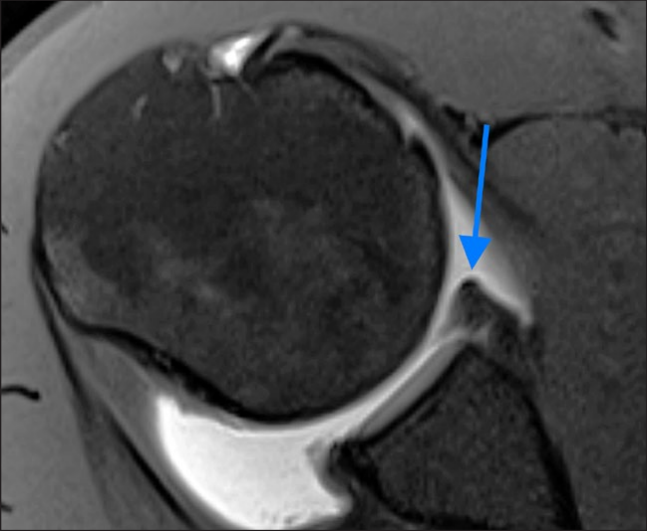
**Bankart lesion.** Axial fat-saturated T1 weighted MR arthrographic image depicts an anteroinferior glenoid labral tear also known as Bankart lesion (arrow) appearing as a labrum ripped off the glenoid rim with irregular inferior outline

**Figure 6:**
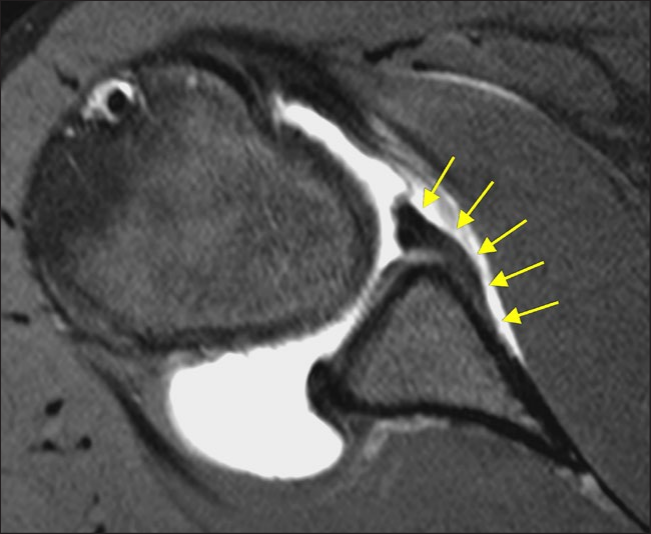
**Perthes lesion.** Axial fat-saturated T1 weighted MR arthrographic image demonstrates a pathological variant of Bankart lesion including a torn undisplaced labrum paired with a medially stripped but intact periosteal sleeve (small arrows outline the torn labrum and the periosteal sleeve).

**Figure 7:**
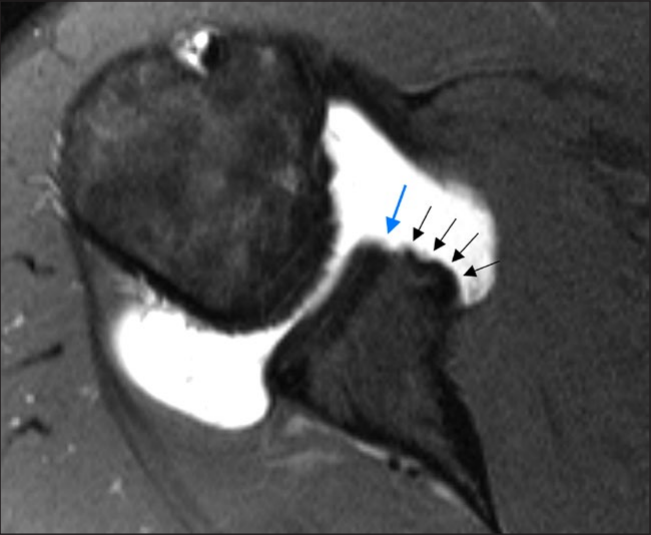
**ALPSA lesion (Anterior labroligamentous periosteal sleeve avulsion).** Axial fat-saturated T1 weighted MR arthrographic image displays a chronically torn anteroinferior labrum, avulsed off together with a periosteal sleeve, which is bunched up medially as a fibrous scarred nodule (black arrows). Note the defect at the location of the normal attachment site of the labrum anteroinferiorly (blue arrow).

The most common lesion of anterior shoulder dislocation, occurring in almost half of the cases, is a Bankart lesion (Figure 5), where the anteroinferior labrum is ripped off the glenoid rim together with the inferior glenohumeral ligament. Many pathological variants have been described in patients with recurrent instability, including a torn labrum with a medially stripped, but intact periosteal sleeve. When the labrum remains (almost) undisplaced in an acute setting, this constitutes a Perthes lesion (Figure 6) (also termed acute ALPSA-lesion, anterior labrum periosteal sleeve avulsion). In chronic cases, the labrum scars in a medially displaced position (ALPSA-lesion, 14%) (Figure 7).

**Figure 8:**
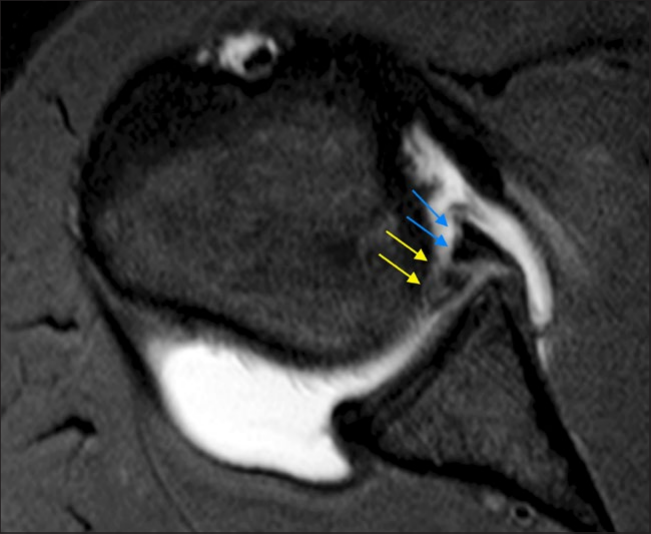
**GLAD (Glenolabral articular disruption).** It is a combination of labral tear (blue arrow) and a piece of adjacent cartilage (yellow arrow) that has been stripped off the glenoid articular surface. It predisposes the shoulder joint to development of osteoarthritis.

Shoulder instability often results in chondral lesions. Loosening of chondral flaps may lead to loose bodies. These chondral injuries predispose to the development of osteoarthritis. Limited cartilage wear and smaller lesions may go undetected on imaging. Multidetector Computed Tomography Arthrography (MDCTA) may be more accurate than MRA for the detection of lesions with substance loss due to a higher sensitivity and specificity. CTA has a higher resolution and slice thickness than routine MRA, as well as a better contrast between the injected contrast material and the grey background. On intermediate and T2-weighted MR arthrographic images, many areas of high signal intensity are seen, including native joint fluid and blood vessels. A specific entity of cartilage damage is the glenolabral articular disruption (GLAD). This GLAD-lesion (Figure 8) is a combination of a superficial labral tear with a piece of cartilage that has been stripped off the glenoid articular surface. Diagnosing cartilage lesions (preoperatively) may help in clearing (postoperative) prognosis of instability treatment as these may predispose to or be the first signs of the development of osteoarthritis.

Perthes/ALPSA-lesions and chondral defects may be difficult to detect. A Perthes lesion may resemble a normal labrum, especially when no contrast is used. An ALPSA-lesion may be mistaken for a blob-like, thickened labrum and type II or III capsular attachment with a medially anchored capsule. As both types of lesion are associated with instability and recurrent dislocation, they are important to recognize. The use of intraarticular contrast is mandatory not to miss these entities. Although not routinely used, two specific positions of the arm that increase the width of a tear have been described. Traction on the labrum and inferior glenohumeral ligament in the ABER position (abduction of at least 90° with external rotation) increases the accuracy of MRA in case of an anteroinferior labral tear with minimal displacement. For an ALPSA-lesion, the FADIR position (full adduction with internal rotation) has been reported to help detection [35,67,68,69,70,71,72,73,74,75,76,77,78,79,80,81,82,83,84,85,86,87,88,89].

### Bony lesions: a bipolar problem not to be underestimated

In a bony Bankart lesion, a piece of the anterior and inferior bony margin of the glenoid has been knocked off together with the labrum. In an acute setting, the size of this bony fragment determines whether the patient can be managed conservatively or should be operated on to fix the fragment. In chronic cases, recurrence may be due to a large bony deficit, either due to erosion of the glenoid rim from repetitive dislocations or because of nonunion or malunion of a large, displaced bony Bankart fragment. Again, accurate determination of the size of the deficit is essential for surgical planning. Smaller bony gaps can still be managed with a soft-tissue repair (‘Bankart procedure’). Larger defects representing > 6 to 8 mm, or > 20 to 25%, of the inferior glenoid diameter require a bony procedure (e.g. “Latarjet procedure”).

**Figure 9:**
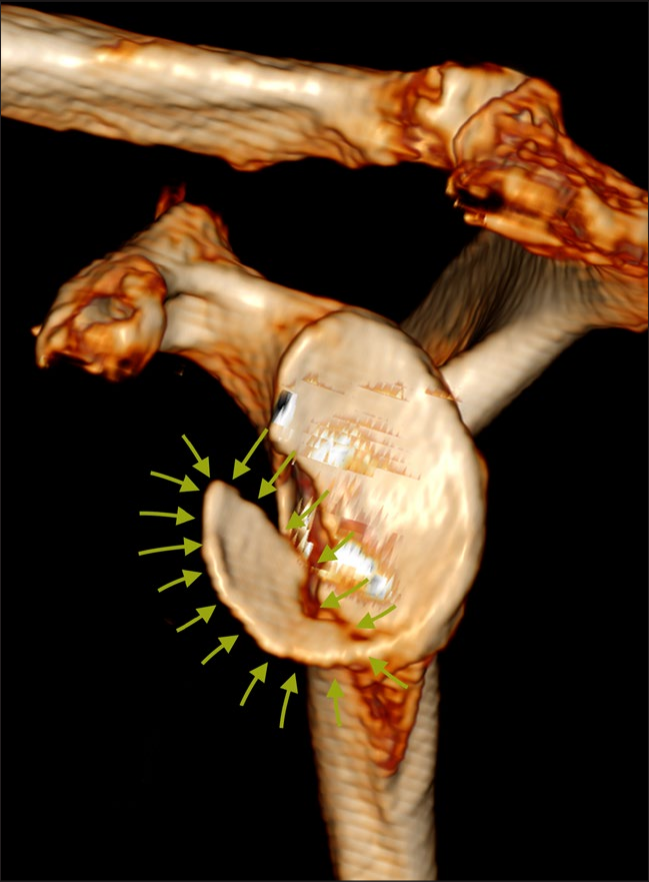
**3D MDCT of bony Bankart lesion.** 3D volume rendering CT of a large avulsed anteroinferior glenoid bony fragment (outlined by small arrows) using surface rendering and subtraction of humeral head that will be treated by surgical fixation.

Several techniques have been reported to gauge the defect. Although sagittal oblique MR images may be used, the reference method is 3D MDCT with sagittal oblique reformatted images or 3D surface rendering with subtraction of the humeral head (Figure 9). One method assesses the anteroinferior surface area loss in comparison with a best-fit circle of the inferior two-thirds of the glenoid face. A vertical line is drawn along the deficient glenoid parallel to the 12 to 6 o’clock axis. Every 1.5 to 1.7 mm glenoid bone loss corresponds approximately with a 5% increase in surface area. Another method uses a percentage of the width of the glenoid based on a horizontal line through the center of the best-fitting circle. A third estimation method uses the (vertical) length of the glenoid as a reference. The vertical height of the defect should not surpass 21% for stability to be maintained. When the glenoid resembles an inverted pear instead of the normal appearance with a wider inferior and smaller superior part, the inferior bone loss can be estimated to be at least 25%.

**Figure 10:**
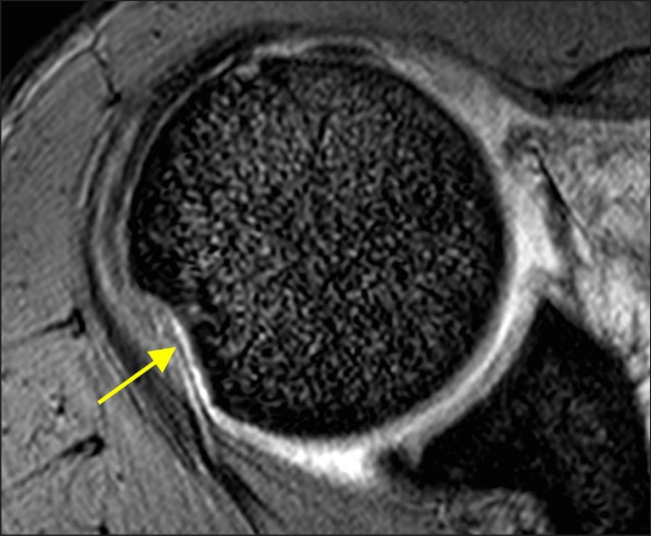
**Hill Sachs lesion.** Axial gradient echo image through superior part of the humeral head shows Hill-Sachs lesion as a smooth impression on its posterior aspect (arrow).

A Hill-Sachs impression (Figure 10) on the posterosuperior aspect of the humeral head is an almost ubiquitous finding of shoulder dislocation (80 to 100%). This lesion can range from a focal region of bone marrow edema on MRI to a large defect of more than a quarter of the humeral head. It only has clinical significance when it reaches sufficient size to engage on the glenoid rim. In that case, the humeral head catches on the glenoid rim and is levered into dislocation. Typically, an engaging Hill-Sachs lesion has a long axis parallel to the anterior rim of the glenoid when the shoulder is in abduction of 90° with external rotation of more than 30° (ABER). When the lesion runs more diagonally and thus not parallel with the anterior glenoid in the ABER position, it will not engage. Engagement will occur with smaller Hill-Sachs lesions when associated with a bony Bankart lesion that diminishes the glenoid width. This principle has been dubbed the ‘glenoid track concept’. Therefore, bony Bankart and Hill-Sachs lesions should be evaluated as a bipolar problem. The size, depth, and orientation of the Hill-Sachs lesion can best be determined on 3D surface rendering of the humeral head. The Hill-Sachs lesion should not be confused posterosuperiorly with the bare area at the junction between the footprint of the infraspinatus and the articular cartilage nor posteroinferiorly with the flattened area of the normal humeral neck. On axial images, the Hill-Sachs lesion is typically found at the level of the coracoid process, whereas the neck is far below this level [1,35,68,72,77,80,84,85,90,91,92,93,94,95,96,97,98,99,100,101,102,103,104,105,106,107].

### Other lesions

When finding a typical Bankart lesion, the pitfall is disregarding the other structures. Identifying additional labroligamentous lesions is paramount since they should be addressed surgically at the same time as the Bankart lesion to avoid failure. As they are more difficult to discern, a high degree of suspicion is necessary.

#### Midsubstance lesions and humeral avulsions

**Figure 11:**
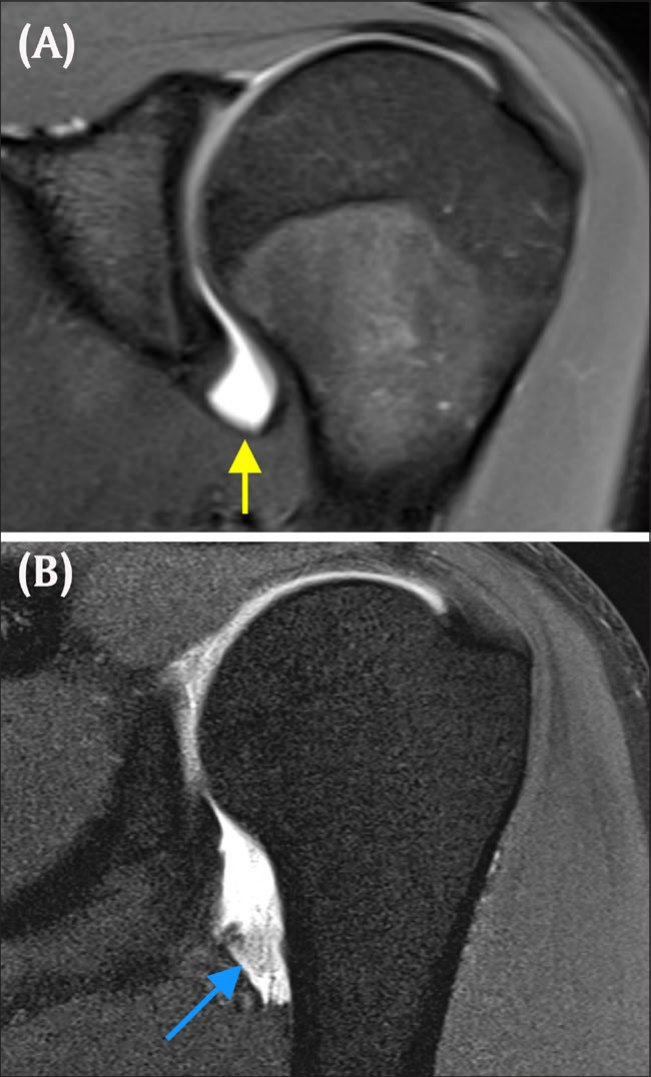
**Normal IGHL and HAGL.** Coronal fat-saturated T1 weighted MR arthrographic images. **A.** Note the smoothly outlined U-shaped configuration (arrow) of normal inferior glenohumeral ligament (IGHL). **B.** Compare the thickened irregular wavy outline of the torn IGHL and conversion of U-shaped axillary pouch to J-shaped structure (arrow) in HAGL (humeral avulsion of inferior glenohumeral ligament).

In up to 10% of patients with anterior instability, damage to the glenohumeral stabilising structures will be on the humeral side. These HAGL-lesions (humeral avulsion of the glenohumeral ligaments) are frequently missed on CTA/MRI/MRA. A torn inferior GHL may appear as a thickened, wavier, irregular structure. The main diagnostic criterion for a HAGL is the conversion of the normal fluid distended U-shaped axillary recess into a J-shaped structure with extravasation of fluid and altered signal intensity on coronal oblique images (Figure 11). False positives may result from a variant V-shaped attachment of the inferior humeral capsule and due to iatrogenic extravasation. False negatives can also occur when scar tissue connects the avulsed ligament to the humeral neck. In cases without the typical J-sign, discontinuity on contiguous slices, altered signal intensity, thickening, waviness and irregularity can be clues for abnormality. When associated with a bony fragment (BHAGL, bony humeral avulsion of the GHL), detection is easier. They may occur in isolation or in association with midsubstance tears or elongation of the glenohumeral ligaments as well as labral lesions. In these instances, the line of tearing crosses the joint from medial to lateral. For example, a posterior HAGL can be seen together with an anteroinferior Bankart and a rotator interval or biceps pulley lesion.

Occasionally, a midsubstance tear or elongation of the anterior band of the inferior GHL (ABIGHL) may occur with or without labral damage. A normal ABIGHL arises from the inferior half of the glenoid labrum. A higher origin is seldomly observed and rarely, the ABIGHL arises from the MGHL. More laterally and at the humeral insertion, the ABIGHL is best evaluated on sagittal oblique images. Coronal oblique images are less useful as they rarely depict the ligament over its length. The ABIGHL is usually well visualized on axial images medially and in its midportion. The ABIGHL can be traced along its length on subsequent axial images as a round to triangular shaped structure of intermediate-to-low signal intensity. Due to partial volume effect, a small area of increased signal intensity may be seen on axial T2-weighted MR images in the ABIGHL near the labrum.

#### Extension to other areas

**Figure 12:**
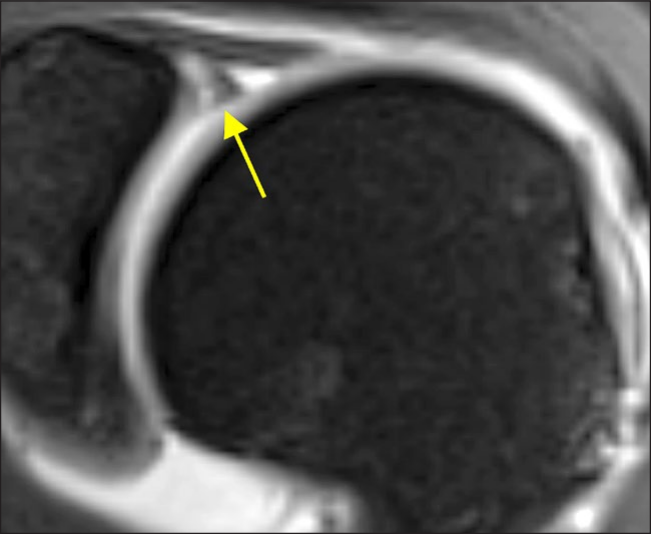
**Type III SLAP tear (Superior labrum from anterior to posterior).** Coronal fat-saturated T1 weighted MR arthrographic image shows type III SLAP tear, a bucket handle type of tear of the bicipitolabral complex (arrow).

**Figure 13:**
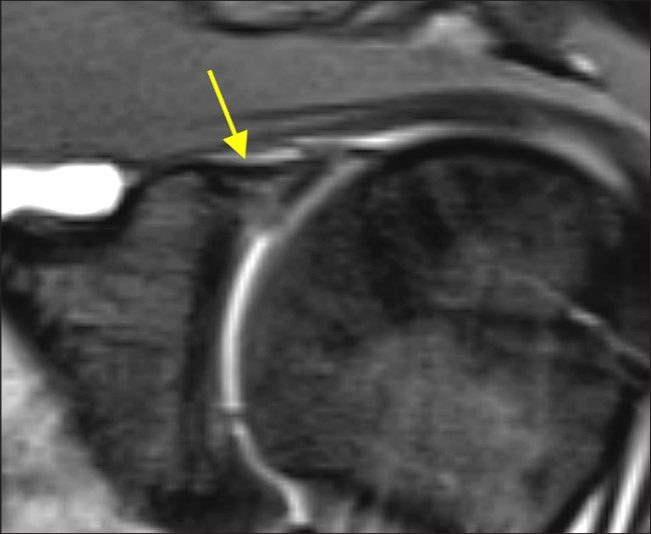
**Type IV SLAP tear.** Coronal fat-saturated T1 weighted MR arthrographic image demonstrates a SLAP tear in which the superior labral tear extends into the biceps tendon (arrow).

In more severe trauma and with increasing numbers of recurrent dislocations, the glenohumeral damage may extend beyond the area of the anterior band of the inferior glenohumeral ligament. Extension superiorly, towards the anterosuperior labrum, including the middle glenohumeral ligament, is common. The superior glenohumeral ligament as well as the superior labrum, including the long tendon of the biceps (SLAP-variants) (Figures 12 and 13), may also be involved. Differentiating an avulsed anterosuperior labrum from a sublabral hole or a Buford complex may be difficult. Posterior extension in a patient with anterior shoulder instability may be limited to the formation of a partial tear along the glenoid rim, but may also be a frank avulsion of the posterior labrum.

Posterior instability may occur independently and can sometimes be difficult to diagnose clinically. It may present with pain only, without clear symptoms and signs of dislocation. On imaging, a posterior counterpart of Bankart, Perthes and ALPSA-lesions can be found. These reversed lesions have the same imaging characteristics as their anterior homonyms. Anteriorly, a reversed Hill-Sachs lesion can be found medial to the lesser tuberosity [9,30,46,67,72,74,95,108,109,110,111,112,113,114,115,116].

### Rotator cuff tears

Especially in patients over 40 years of age, anterior shoulder dislocation may occur in conjunction with tears of the rotator cuff tendons. These may be acute or chronic and can be partial or complete. Most often, the supraspinatus tendon is involved, but the subscapularis and infraspinatus can be implied as well. However, several studies have shown that partial or full-thickness tears may occur in asymptomatic patients, even in up to 52% of high-level throwing athletes. These lesions may therefore have been pre-existing and not require treatment [16,18,72,95,117,118].

## Anterosuperior instability

### Normal and variant anatomy and imaging

The rotator interval (RI) is defined as the triangular area between the anterior border of the supraspinatus tendon and the superior border of the subscapularis tendon, ranging from the coracoid process to the biceps groove. At the capsuloligamentous level, the roof of this space is formed by the anterior part of the superior complex, i.e.: the superior GHL (SGHL) and coracohumeral ligament (CHL). The inferior border of the RI is formed by the middle GHL.

The SGHL can be seen to arise from the superior labrum, just anterior to the insertion of the long tendon of the biceps at the level of the base of the coracoid process. The origin can also be from the biceps tendon instead of from the labrum or in common with the MGHL. The SGHL courses almost parallel with the coracoid process, nearly perpendicular to the MGHL in neutral rotation. Therefore, it can well be visualized on axial images, where it appears as a thicker, anteriorly convex band anterior to the long tendon of the biceps. On sagittal oblique images, the SGHL normally appears as a thin, almost horizontally and anteriorly oriented band, just inferior to the CHL and coracoid process. Occasionally, a normal SGHL can appear thicker and may then be associated with an absent or vestigial MGHL. The coracoglenoid ligament (CGL) appears as a straight thin line extending from the superior glenoid rim to the coracoid process on axial and sagittal images. In the midportion of the rotator cuff interval, the CHL can readily be identified on imaging as a flat structure with homogeneous, low signal intensity when the joint is fluid-filled.

Laterally, the CHL and SGHL merge to form a complex that cradles the long tendon of the biceps anteroinferiorly. It can be identified as a T-shaped structure interposed between this tendon and the subscapularis in a fluid-filled joint. Even a thin SGHL can often be visualized on thin slices as a structure of slightly higher signal intensity than the subscapularis before inserting in a small depression on the humeral head (the fovea capitis). The CHL merges with the rotator cable to cover the long tendon of the biceps posterosuperiorly before inserting on the greater tubercle around the bicipital groove.

The MGHL has the greatest variation in size and attachment of all glenohumeral ligaments. It is best visualised on axial or sagittal oblique images. Medially, the MGHL is seen as a hypointense structure arising from the anterosuperior part of the glenoid labrum or neck. This origin can be conjoint with the origin of the SGHL or, less frequently, with the origin of the long tendon of the biceps superiorly or the origin of the anterior band of the IGHL (ABIGHL) inferiorly. The midportion of the MGHL usually appears as a flat band on sagittal oblique images, just posterior to the subscapular tendon. On axial images, the MGHL usually appears in a more or less lengthened cross-section of a longitudinal band. Depending on the degree of humeral rotation, the MGHL may appear thicker and wavy (with more internal rotation) or thinner and straighter (with more external rotation). Contiguous axial slices should be examined to follow the MGHL from medial to lateral and to avoid confusion with a capsulolabral detachment, especially in the case of a cordlike MGHL. Often, the majority of its anteroinferior oblique course can be visualized on sagittal oblique images of a fluid-distended joint. Coronal oblique images rarely show the MGHL, because of partial volume effect with the anterior shoulder capsule and subscapular tendon, unless it is folded up or thickened. Laterally, the MGHL becomes difficult to differentiate as it blends with the subscapular tendon and the fasciculus obliquus (FO). In case of a cordlike MGHL, it will appear rounded on axial images and thickened in the sagittal oblique plane. A Buford complex, the absence of the anterosuperior labrum next to the cross section of a thickened MGHL on axial images, should not be confounded with a labral tear. The thickened, cordlike MGHL seen in a Buford complex is an example of a glenoid labrum ovoid mass (GLOM). A double MGHL will be seen as a double parallel structure on sagittal oblique images and as a U-shaped structure on axial images. This variant should be differentiated from a labral tear.

The rotator interval can best be appreciated on sagittal oblique images. With intraarticular contrast, the normal anterosuperior capsule from SGHL to MGHL should appear as a curved, smooth area with a thickness of about 2mm just lateral to the coracoid process [9,119,120] [1,11,28,81,113,121,122,123,124,125,126,127,128].

### Anterosuperior instability: Rotator interval, MGHL and SGHL pathology

An elongated and/or (partially) torn middle or superior glenohumeral ligament results in an increased rotator interval. Isolated lesions of these two ligaments with or without labral involvement are more frequently associated with anterosuperior instability. This type of instability often does not present with an unstable shoulder, hence the name minor or occult instability. Frequently, these patients have a history of hyperlaxity and overhead sports. Repetitive micro-trauma may be a cause of progressive wear and tear. The clinical examination can be difficult due to pain and often the clinical diagnosis is based more on a high degree of suspicion than on specific findings. Interpretation of imaging is made difficult by the many normal variants that can occur in this area. Because of its better soft tissue delineation, higher field strength MRA may be more helpful than CTA.

Distension, increased volume and irregularity of the interval or extra-articular contrast material may help in raising suspicion. Signs of rotator interval (RI) lesions on imaging include irregularity of the normally smooth convex contour of the SGHL on the sagittal oblique images, discontinuity as well as penetration of contrast material that extends to the undersurface of the coracoid process displacing the normal extracapsular fat. The rotator interval, however, is prone to inflammation with development of synovitis. This may also result in an obscured appearance of the RI. Other signs to look out for in the ligaments are waviness, contrast leakage, altered signal intensity, changes in the expected calibre and course. A distended RI may be associated with thinning of the involved ligaments, however, thickening of CHL and/or SGHL can also be observed. With a distended RI, the SGHL adopts a course that lies more in the axial plane and the midsection of the MGHL will drop more inferiorly. For the MGHL, discontinuity on contiguous sagittal oblique and axial images, especially when associated with waviness and thickening, is another reliable sign of abnormality. An elongated or partially torn MGHL may be suspected when it displays a more horizontal course in neutral rotation. This will result in a longer cross-section on axial images.

At the medial side, tears of the anterosuperior labrum between 12 and 3 o’clock have to be differentiated from the normal variants of this area (sublabral hole, Buford) and SLAP-lesions from sublabral sulci. On the other hand, a sublabral sulcus may predispose to tearing of the labral attachment right next to the hole. Likewise, a thin or rudimentary middle glenohumeral ligament may be more easily distended. On the humeral side, an avulsion of the middle glenohumeral ligament can be mistaken for, but also associated with, a partial tear of the subscapularis tendon [9,74,122,129,130,131,132,133,134,135,136,137,138,139].

### Biceps pulley lesions and biceps instability

**Figure 14:**
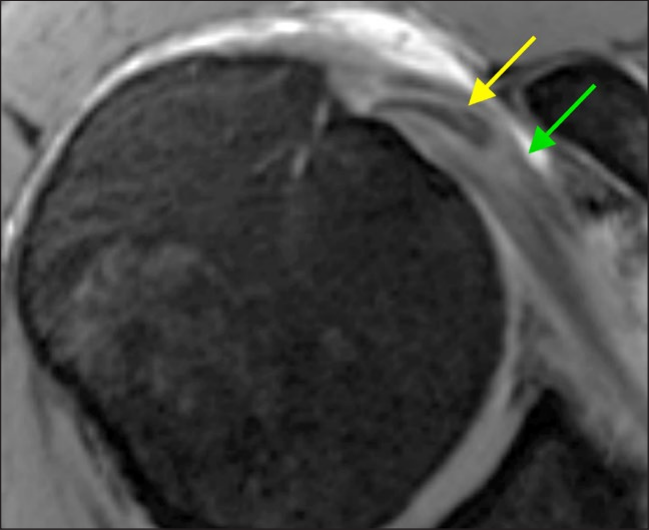
**Biceps tendon instability.** The long head of the biceps tendon is stabilized by the biceps pulley. Damage to the biceps pulley can cause biceps instability and medial displacement of the biceps tendon. On this axial fat-saturated T1 weighted MR arthrographic image, the long head of the biceps tendon is displaced medially (yellow arrow) and is found lying within the subscapularis tendon which presents an increased signal intensity (green arrow).

The humeral insertion of the superior glenohumeral ligament forms the inferior part of the biceps pulley. Damage here may lead to medial biceps instability (Figure 14). Accurate imaging is very important as this type of biceps instability can easily be missed intraoperatively because the biceps tendon is not dislocated intraarticularly. When the biceps pathology is not addressed appropriately, this may lead to failure of treatment. Diagnostic criteria for biceps pulley lesions include subluxation or dislocation of the biceps tendon relative to the subscapularis tendon on oblique sagittal images or medial subluxation on axial slices, although an unstable biceps tendon may appear centered with the arm in neutral rotation. Tearing of the superior border of the subscapularis and nonvisibility or discontinuity of the lateral superior glenohumeral ligament appear to be quite accurate signs, although an intact border of the subscapularis does not exclude a pulley lesion. Signs of biceps tendinopathy on sagittal oblique images (caliber changes, abnormal signal intensity), tears of the coracohumeral ligament and tears of the anterior fibers of the supraspinatus seem to be less sensitive. The tendon may also appear flattened or thickened within the interval or in the intertubercular groove. Signal changes in the intrarticular part of the biceps tendon are nonspecific due to the magic angle effect and partial volume averaging [82,129,140,141,142,143,144].
